# A qualitative focus group study on legal experts’ views regarding euthanasia requests based on an advance euthanasia directive

**DOI:** 10.1186/s12910-024-01111-2

**Published:** 2024-10-24

**Authors:** D. O. Coers, S. H. Scholten, M. E. de Boer, E. M. Sizoo, M. A. J. M. Buijsen, B. J. M. Frederiks, C. J. W. Leget, C. M. P. M. Hertogh

**Affiliations:** 1 Department of Medicine for Older People, Amsterdam University Medical Center, De Boelelaan 1117, 1081 HV Amsterdam, The Netherlands; 2 Amsterdam Public Health Research Institute, Aging & Later Life, Amsterdam, The Netherlands; 3 Erasmus School of Health Policy and Management, Erasmus School of Law, Erasmus University Rotterdam, Burgemeester Oudlaan 50, 3062 PA Rotterdam, The Netherlands; 4 Department of Law & Medical Humanities, Amsterdam University Medical Center, De Boelelaan 1117, 1081 HV Amsterdam, The Netherlands; 5 Amsterdam Public Health Research Institute, Quality of Care, Amsterdam, The Netherlands; 6 Department of Care Ethics, University of Humanistic Studies, Kromme Nieuwegracht 29, 3512 HD Utrecht, The Netherlands

**Keywords:** Euthanasia, Dementia, Legal guidance, Advance euthanasia directives, Decision-making process, Medical ethics, Health law, UN Convention

## Abstract

**Background:**

The Dutch Euthanasia law permits euthanasia in patients with advanced dementia lacking decisional capacity based on advance euthanasia directives. Nevertheless, physicians encounter difficulties assessing the criteria for due care in such cases. This study explores the perspectives of legal experts on the fulfillment of these criteria and the potential for additional legal guidance to support physicians’ decision-making processes.

**Methods:**

A qualitative study was conducted with legal experts. Two focus group sessions were conducted. The data analysis was conducted iteratively, with the data being interpreted using thematic content analysis and the framework method.

**Results:**

Participants emphasize the importance of considering the patient’s current wishes and informing them about the limitations of advance euthanasia directives. While representatives and healthcare professionals can assist in interpreting wishes, the final decision regarding euthanasia rests with the physician. The participants also discuss the challenges posed by pre-recorded wishes due to changing preferences. Furthermore, they present different views on the value of life wishes of patients with advanced dementia. While some participants prioritize life wishes over advance euthanasia directives, others question whether such expressions still reflect their will. Participants find it essential to assess unbearable suffering in the context of the current situation. Participants acknowledge the necessity to interpret advance euthanasia directives but also current expressions and they entrust this interpretation to physicians, viewing them as the primary authority, despite consulting multiple sources.

**Conclusions:**

The Dutch Euthanasia law’s due care criteria are open norms –which are open in substance and require further elaboration, mostly determined on a case-by-case basis to the field standards of the profession–, placing the responsibility on physicians to interpret advance euthanasia directives and patient expressions. Despite potential support from various sources of information, there is limited additional legal guidance available to assist physicians in making decisions.

**Supplementary Information:**

The online version contains supplementary material available at 10.1186/s12910-024-01111-2.

## Background

While the Dutch ‘Termination of Life on Request and Assisted Suicide Act’ (hereafter referred to as the Euthanasia law) [1] permits euthanasia or physician-assisted suicide (EAS) based on a written advance euthanasia directive (AED) (Table 1), physicians face challenges in evaluating the due care criteria in cases of patients with advanced dementia and an AED. These criteria presuppose a communicative relationship with the patient and are solely based on experiences with patients capable of making decisions, rooted in case law. Case law is formed through judicial decisions, creating precedents that guide future rulings and help interpret or fill gaps in legislation. To extend EAS practice beyond these presuppositions and case law to patients lacking decisional capacity, the legislator added Article 2.2, stating that in these cases, the due care criteria must be applied *‘mutatis mutandis’* (Table 1) [2–6]. However, ever since the enactment of the Euthanasia law, the meaning of this formula has been ambiguous to most physicians. Earlier research has shown that physicians report needing at least some sort of ‘meaningful communication’ with the patient in order to be able to perform EAS in patients with dementia and limited decisional capacity [6–9]. Furthermore, the Royal Dutch Medical Association (RDMA) formally stated in 2012 that ‘verbal confirmation of the AED’ was mandatory for physicians to comply with the AED [10]. In their interpretation of the *‘mutatis mutandis’* formula, the RDMA, staying close to the moral foundation of the original due care criteria, attached great value to a confirmation –in word or behavior– by the patient of his AED in order for a physician to be able to comply with the due care criteria. This interpretation of Article 2.2 and the *‘mutatis mutandis’* formula was criticized by the Regional Euthanasia Review Committees (RERC), arguing that requiring confirmation imposes too strict an interpretation of the Euthanasia law, undermining the flexibility intended by the *‘mutatis mutandis’* formula, which should allow for adjustments in cases where communication is impossible. This conflicting criticism has left physicians uncertain about how to apply the due care criteria in cases involving AEDs and advanced dementia.

**Table 1:** The (origin of the) Dutch Termination of Life on Request and Assisted Suicide Act, the statutory due care criteria and Article 2.2 [1, 11]

In the Netherlands, EAS has been regulated in the Euthanasia law since 2002. Though EAS is still a criminal offence, and physicians are released from liability on the condition that the statutory due care criteria are met and that the EAS is reported to a RERC.
**The following statutory due care criteria are included in the law:**
1. *“The physician must be satisfied that the patient’s request is voluntary and well-considered.”* 2. *“The physician must be satisfied that the patient’s suffering is unbearable*,* with no prospect of improvement.”* 3. *“The physician must have informed the patient about their situation and prognosis.”* 4. *“The physician*,* together with the patient*,* must have come to the conclusion that there is no reasonable alternative in the patient’s situation.”* 5. *“The physician must have consulted at least one other*,* independent physician*,* who must see the patient and give a written opinion on whether the due care criteria set out above have been fulfilled.”* 6. *“The physician must have exercised due medical care and attention in terminating the patient’s life or assisting in the patient’s suicide.”*
Article 2.2 of the Euthanasia law states: *“If the patient aged sixteen years or older is no longer capable of expressing his will*,* but prior to reaching this condition was deemed to have a reasonable understanding of his interests and has made a written statement containing a request for termination of life*,* the physician may carry out this request. The requirements of due care*,* referred to in the first paragraph*,* apply mutatis mutandis.”*

In April 2020, the verdict in the first-ever euthanasia case to be brought before a criminal court, and ultimately the Supreme Court, was made public [12–16] (Appendix 1 and 2). In this case, EAS was applied to a patient with advanced dementia without communication with the patient. The decision granted more leeway by eliminating the need to verify the patient’s desire for EAS and giving the physician authority to interpret the AED. However, this change imposes a huge responsibility on the physician without providing clear guidance. Consequently, the Supreme Court’s ruling also led the RDMA no longer to require a mandatory ‘verbal confirmation of wish’ [17].

It is relevant to note that the Supreme Court’s ruling solely relied on Dutch legislative history (Appendix 3) and did not consider a human rights perspective [18–20]. Yet, Article 12 of the United Nations Convention on the Rights of Persons with Disabilities (UNCRPD) [21] states that people with disabilities –including those with decision-making impairments– shall enjoy legal capacity on an equal basis with others in all aspects of life. It emphasizes the importance of providing appropriate support to help them exercise their legal capacity. Article 12 of the UNCRPD [21] aims to shift from substitute to supportive decision-making. This shift entails considering the current wishes and preferences of individuals, as well as any prior declarations. This approach may have significant implications for the practice of substitute decision-making based on an AED in the EAS-procedure [22], as it requires consideration of the actual will of the person at the time of decision-making rather than merely the AED.

In addition to the court’s ruling, various legal and ethical experts have provided their insights on this topic, reflecting considerable variation in the interpretations of Article 2.2 [19, 23, 24]. Some argue for a stringent interpretation where patients must affirm their current death wish as stated in their AED [19, 23]. On the other side of the spectrum, experts state that in patients with advanced dementia who lack decisional capacity, current expressions may not legally revoke an existing euthanasia wish documented in an AED [24].

Considering these developments and conflicting interpretations of Article 2.2, the following research question was formulated: “How can physicians fulfill the statutory due care criteria *‘mutatis mutandis’* if ‘meaningful communication’ can no longer be a criterion to hold on to, and what additional legal guidance, also based on human rights, can be offered to them by legal experts in this regard?”.

## Methods

This focus group study forms part of a comprehensive research project entitled: *“Euthanasia in patients with dementia and an advance euthanasia directive: towards practical guidance” (DALT)*. The overarching objective of the DALT project is to develop a research-based practice guidance for physicians, offering approaches to handling AEDs in patients with advanced dementia. In different parts of this project, all relevant perspectives (from physicians, ethicists, legal experts and patient representatives) are included in the decision-making process. This sub-study focuses on the perspectives of legal experts.

### Design

A qualitative study was conducted with legal experts familiar with the Euthanasia law, Human Rights and expertise on the topic of EAS based on an AED in people with advanced dementia. Two focus group sessions were held to gather data. Based on the literature [2, 6, 7, 25, 26] and other findings of our research [6, 27], a topic list was formed (Appendix 4) and visualized in a triangular figure (Appendix 5). Findings have been presented according to the Consolidated Criteria for Reporting Qualitative Studies (COREQ) checklist [28]. The Medical Ethics Review Committee of Amsterdam UMC, location VU University Medical Center, approved the study protocol (2019.018).

### Participants

Participants were purposefully selected to represent diverse fields of legal expertise, considering their profession, experience and background (Table 2). Despite a limited number of experts on the topic, twenty-one were invited, ensuring equal representation in both focus groups. Experts whose viewpoints are well-established in the literature through earlier articles or opinion pieces were excluded to allow for a broader range of perspectives that have not yet been (extensively) explored, without focusing on whether these views are in favor of or opposed to euthanasia. Participants received written information about the study, including the research question and topic list figure (Appendix 5), and provided informed consent.

### Data collection

Two of the invited legal experts declined participation, six were unavailable during the study period, and two did not respond to the invitation. Prior to the start of the focus groups, one participant failed to attend without prior notification, and another canceled on the scheduled day of the focus group. This resulted in nine legal experts participating in the study. Rather than continuing data collection until data saturation was reached, the limited availability of legal experts forced data collection to be limited to two focus groups with four participants in the first focus group and five in the second focus group. Each focus group was led by an experienced moderator (CJW and CMPMH), who were aware of their own assumptions regarding the research topic. Field notes were taken on non-verbal communication, noteworthy quotations and surprising subjects. All data were pseudonymized. Due to the COVID-19 restrictions, both focus groups were conducted digitally via Zoom [29] and lasted 90 min. The moderators provided a brief introduction to the dilemmas presented in the triangular topic list figure, after which space was provided for open discussion on the topic. Both sessions were audio and video recorded and transcribed verbatim.

### Data analysis

Data analysis was performed iteratively, starting with open double coding of the first transcript, where two researchers (DOC and SHS) independently coded the data using initial categories. This was followed by discussions within the research team (all authors) to reconcile and refine the coding. The codebook was then adjusted based on these discussions. The second focus group was conducted, with topics from the first focus group tested and additional topics introduced by the moderator to address any areas not yet explored in depth. The transcript of the second focus group was also double-coded by two researchers (DOC and SHS), and the codebook underwent further adjustments based on this round of coding.

Ideally, data collection would continue until data saturation was reached. Unfortunately, non-response made it impossible to include more legal experts in these focus groups. Data were interpreted using a combination of thematic content analysis [30–32] and the framework method [33]. The framework method is a systematic and flexible approach for managing and analyzing qualitative data, organizing it into a matrix where rows represent participants and columns representing themes or codes. This matrix allows for a structured summary of data and facilitates the exploration of patterns both within and across cases. MaxQDA (2020) software was used to manage the data [34]. The interpretation of themes was discussed and agreed upon by the research team.

## Results

Nine legal experts participated, four in the first focus group and five in the second focus group (Table 2).

**Table 2:** Participant characteristics

Focus group	Participant	Gender	Field of legal expertise
I	I.I	Male	Health law
	I.II	Male	International Human Rights, criminal law
	I.III	Female	Health law
	I.IV	Male	Health law
II	II.I	Male	Health law
	II.II	Male	Health law
	II.III	Female	Family law
	II.IV	Female	Health law, International Human Rights
	II.V	Male	Notary law

**Fig. 1:**
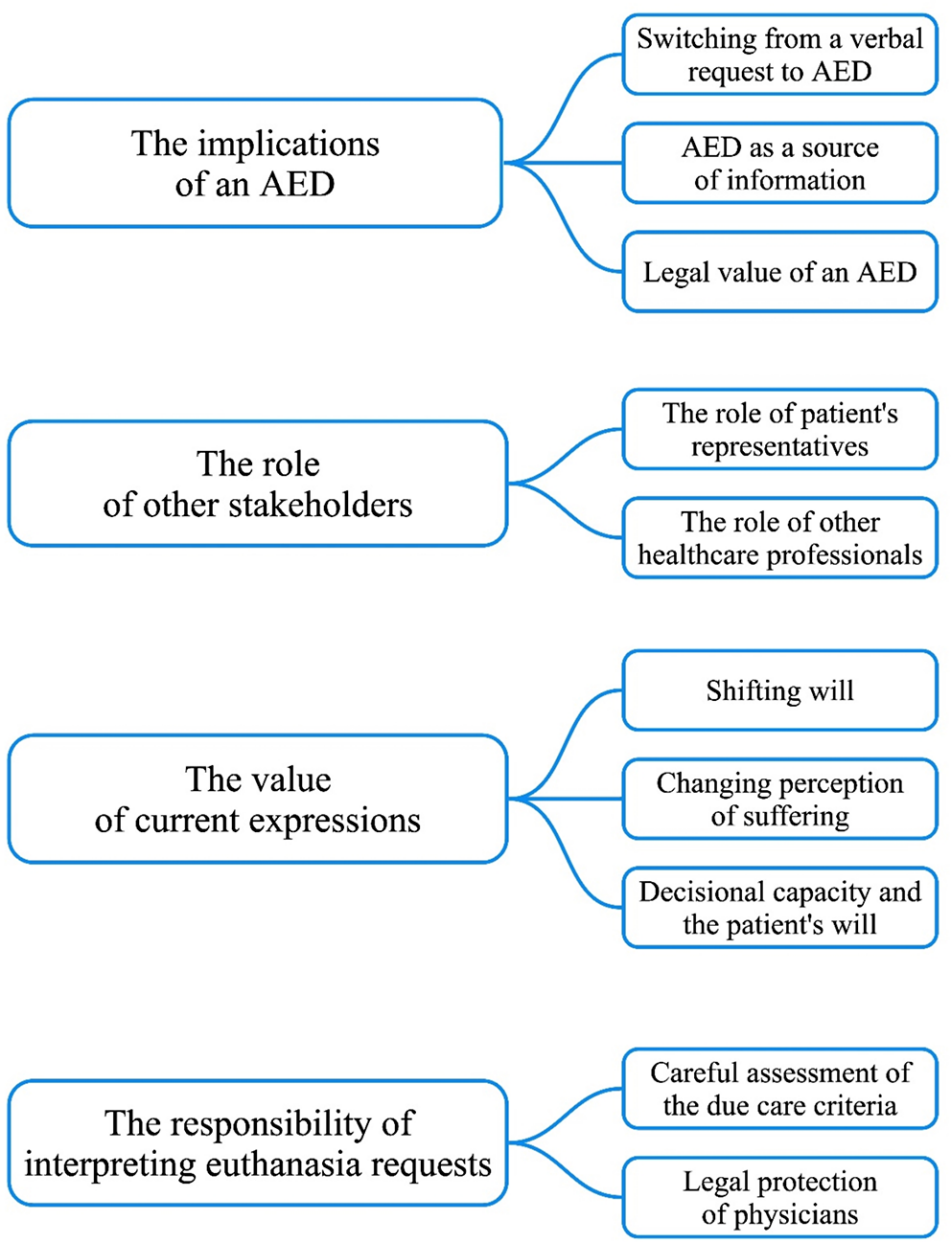
Themes emerging from the data

Four main themes emerged from the data: (1) the implications of an AED, (2) the role of other stakeholders, (3) the value of current expressions and (4) the responsibility of interpreting euthanasia requests (Fig. 1).

### The implications of an AED

#### Switching from a verbal request to AED

Participants acknowledge the role of the AED under the Euthanasia law (Table 3, q1.1). However, it is crucial to first determine decisional capacity in the context of EAS before considering an AED as a replacement for an oral request (Table 3, q1.2). Patients expect that the AED will be used when they can no longer request EAS themselves; in other words, they expect it to substitute their voice in decision-making. Nonetheless, it was said that according to the UNCRPD, only supportive decision-making is conceivable in the case of EAS (Table 3, q1.3). As long as the patient can articulate their current wishes, those wishes should be explored since the AED primarily considers the patient’s wishes at the time of writing (Table 3, q1.4). Additionally, the importance of assessing decisional capacity at the time of writing is stressed (Table 3, q1.5), without this being a mere box-ticking exercise (Table 3, q1.6).

#### An AED as a source of information

Two sorts of information can be derived from patients’ AED: information on the desired time of execution, frequently linked to (fear for) a specific form of suffering, and information about patients’ viewpoints on (unbearable) suffering (Table 3, q1.7).

#### Legal value of an AED

The idea that a euthanasia request is settled by drawing up an AED is misleading. Regular conversations with a physician are needed to keep the request up-to-date and relevant (Table 3, q1.8), although the capabilities of patients with declining cognitive abilities to have such conversations are doubted (Table 3, q1.9). Patients should be informed about the relativity of AEDs, especially considering the complexity of predicting the future (Table 3, q1.10 and q1.11). Some participants request a better, neater phrasing of the AED in an attempt to prevent interpretation problems that could arise from ambiguous or unclear language (Table 3, q1.12). Certain others share the view that inconsistencies in the AED’s text should be avoided, yet they simultaneously acknowledge that complete avoidance may not be feasible (Table 3, q1.13). Suggested as potentially beneficial is to strongly recommend or even require patients to be advised by a legal expert when drafting their AED (Table 3, q1.14). The existing AED formats serve as mere examples; nevertheless, patients should be advised to include a personal supplement (Table 3, q1.15). Opinions vary regarding the level of precision in wording. Some participants believe patients should express their desires in a personalized and detailed manner to ensure their intentions are accurately captured (Table 3, q1.16), while others prefer a more general approach. They believe that using broader and less specific language will result in fewer legal inconsistencies or potential conflicts when interpreting the patients’ wishes (Table 3, q1.17).

### The role of other stakeholders

Although representatives of the patient (such as a legal representative or family member) and other involved healthcare professionals (such as other physicians, nurses, psychologists, and spiritual counselors) can function as sources of information, the physician is said to be responsible for the final decision in the EAS process (Table 3, q2.1).

#### The role of patient’s representatives

The role of representatives in the decision-making process is defined as limited (Table 3, q2.2). Their help is needed in interpreting patients’ wishes and expressions, but they can never make the final decision (Table 3, q2.3 and q2.4). In cases where conflicts arise in interpreting the patient’s AED between the physician and the patient’s representative, the representative’s arguments should not be disregarded (Table 3, q2.5). It is suggested that conflicts of interpretation between patient representatives regarding the patient’s will can potentially be resolved through consultation with a colleague (Table 3, q2.6). In addition, attention must be paid to possible influencing of the will (‘undue influence’) (Table 3, q2.7) or conflicting interests of the representatives (Table 3, q2.8). Therefore, participants advise patients to assign a representative in their AED (Table 3, q2.9) and ascertain that this representative is adequately informed (Table 3, q2.10).

#### The role of other healthcare professionals

Participants confirm the valuable information other involved healthcare professionals can offer. Their input should not be set aside but is presumably already used in clinical practice (Table 3, q2.11).

### The value of current expressions

Patients’ wishes can change over time, and it is, therefore, difficult to record explicit wishes in advance in an AED. Additionally, questions arise about how to interpret the (non-)verbal expressions of a patient with severe dementia.

#### Shifting will

Based on Human Rights Treaties, it is important to determine the patient’s current *“own will and preferences”* (Table 3, q3.1) since the formerly recorded wishes are only of relevance when the patient’s current wish cannot be ascertained (Table 3, q3.2). On the other hand, other participants argue that a patient’s ability to express their will diminishes during the dementia process, making the verification of the will legally complicated (Table 3, q3.3). Besides, some participants consider including Human Rights Treaties in the decision-making process to be an excessive demand on physicians (Table 3, q3.4). This expectation refers to the need for physicians to take into account not only national laws, such as the Euthanasia Law or Supreme Court rulings, but also international frameworks like Human Rights Treaties. Participants expressed concern that requiring physicians to consider these broader legal obligations could add significant complexity to their decision-making processes, making their role more challenging. Therefore, they state that as long as physicians act within the scope of the Euthanasia law, no human rights are violated (Table 3, q3.5).

#### Changing the perception of suffering

Participants argue that the degree of unbearable suffering should be assessed in the present. The former expectations of unbearable suffering play a limited role (Table 3, q3.6), as sometimes there appears to be no suffering, even though the situation described in the AED, which the patient perceives as unbearable suffering, occurs (Table 3, q3.7). Besides, hypothetical situations of suffering should be discussed in more detail before drafting up the AED (Table 3, q3.8).

#### Decisional capacity and the patient’s will

It was said that irreversible decisions leading to death, such as EAS, require a higher threshold for competence (Table 3, q3.9). Still, participants also highlight the challenge of verifying patient’s wishes for EAS, especially when facing progressive dementia (Table 3, q3.10). On the other hand, it was also stated that patients’ expressed wish for life is so fundamental and almost instinctive that it should take precedence over an AED, considering the patient competent in the matter to express their *“own will and preferences”* now (Table 3, q3.11).

### The responsibility of interpreting euthanasia requests

Judging whether a case meets the due care criteria is an interpretative process. It was suggested that much comes down to interpreting the patient’s will rather than establishing it (Table 3, q3.12), and the interpretation is thus subject to the subjectivity of the listener (Table 3, q3.13). Furthermore, participants argue that the interpretation of a patient’s AED or their current (non)verbal expressions is beyond the knowledge and competence of a legal expert (Table 3, q4.1). Therefore, some of the participants place the responsibility for this interpretation with the physician (Table 3, q4.2). While the participants are well aware that the physician must consult various sources to reach a final decision, a widely shared argument is that the physician is seen as the *“master of interpretation”* in the decision-making process (Table 3, q4.3).

#### Careful assessment of the due care criteria

From a legal perspective, the judgment of whether the due care criteria are fulfilled is relatively straightforward, as EAS cases are reviewed in hindsight within the legal framework of the Euthanasia law (Table 3, q4.4). The physician must be able to carefully substantiate their decision-making process (Table 3, q4.5), within the leeway provided by the law. Caution is advised to physicians in case of doubts about the voluntariness or thoughtfulness of the request (Table 3, q4.6). Regarding the decision-making process, some participants suggest using medical professional standards as a potential guide for physicians (Table 3, q4.7). However, it was also emphasized that these guidelines should allow for flexibility, accommodating physicians’ personal assessment and contemplations (Table 3, q4.8).

#### Legal protection of physicians

The legal protection of physicians to make these kinds of complex decisions is stressed (Table 3, q4.9). This protection aims to shield physicians when providing EAS based on an AED for patients with dementia, ensuring that such actions are not considered a patient’s right or a physician’s duty under the law. However, there are concerns about how well this protection is perceived and understood by physicians (Table 3, q4.10).

**Table 3:** Quotations of the participating legal experts

Quote	Participant	Quotation
***The implications of an AED***		
q1.1	pI.V	*‘…That ultimately*,* when it becomes an issue*,* such a request made in the past is one of the elements of the due care criteria that must be taken into account.’*
q1.2	pI.III	*‘My point is that it starts with a written advance directive. […] But that’s preceded by the determination of whether someone’s actually incompetent in this matter. […] So that’s about someone no longer being able to determine their current will regarding their decision of whether or not to want euthanasia.’*
q1.3	pII.III	*‘I’m also looking at the UN Disability Convention [United Nations Convention on the Rights of Persons with Disabilities]*,* and I think that older people predict [expect] substitute decision-making*,* but I believe that when it comes to euthanasia*,* only support[ive] decision-making is conceivable.’*
q1.4	pII.III	*‘So long as someone can express their will*,* by themselves*,* this advance directive is just a ‘collateral.’ You can say something about how you thought about it at the time*,* but you have to look into it further*,* because… how someone thinks about it now.’*
q1.5	pI.II	*‘When you look at the text of the law*,* you also see that the assessment of a reasonable valuation of interests must take place when the advance directive is drawn up. And not in retrospect.’*
q1.6	pI.III	*‘Especially on this very subject*,* I would say: avoid checklists at all times. The physician must be able to explain thoroughly and independently why he or she has come to his or her decision.’*
q1.7	pII.II	*‘…is that two things are often included in requests: when should it take effect? And that’s linked to situations in which people say that the suffering is no longer acceptable suffering. Like: I find it unacceptable to go to a nursing home*,* that would be great suffering for me*,* I shouldn’t end up there…’*
q1.8	pI.I	*‘Those people go to a civil-law notary and they leave the office thinking that everything’s been arranged*,* but that’s not true. You actually have to continuously talk and refresh as much as possible. Then it will remain a useful document.’*
q1.9	pI.II	*‘I have some scepticism about the idea that people in a situation of declining cognitive abilities would be able to provide an update every time that would improve the originally given advance directive.’*
q1.10	pII.V	*‘So*,* the relativity of all that you may sign in terms of notarial instruments… its meaning must also be made clear. To the client too… primarily to them even.’*
q1.11	pI.II	*‘It’s very difficult to foresee what circumstances may occur.’*
q1.12	pI.I	*‘Which leaves room for interpretation*,* but you should try to prevent this as much as possible. […] Yes*,* there’s something to be gained from better*,* neater phrasing. Avoiding interpretation problems.’*
q1.13	pI.III	*‘That you can’t make watertight whatever might be the patient’s wish at some point in the future. And what exactly they want and don’t want. But I particularly advocate clarity to avoid inconsistencies.’*
q1.14	pI.I	*‘That you […] may make it mandatory or strongly recommend that you are advised by a legal professional. […] someone who also takes a look at it.’*
q1.15	pI.III	*‘You could give tips*,* for example. […] Or an explanation of the example document: this is an example; you still have to think very carefully about actual situations… about what you would want or not.’*
q1.16	pII.I	*‘A standard text… you’d better be advised to describe in your own words what unbearable suffering means to you. That’s not apparent here [general statement shared in chat]. […] Yes*,* if that isn’t recorded in further detail*,* it will still be unclear.’*
q1.17	pI.II	*‘That I would prefer [a generic directive] over a detailed advance directive that attempts to address all kinds of circumstances that are difficult for people to foresee.’*
***The role of other stakeholders***		
q2.1	pI.IV	*‘But when it comes to the difference in perspective between the representative and the physician*,* I am more inclined to put the primacy in the hands of the physician. Admittedly*,* I listen […] to various sources. When it comes to family*,* nurse and representative. But he [the physician] really has to support it.’*
q2.2	pI.II	*‘They [representatives of the patient] can serve as a resource for the physician*,* but they have no voice themselves. Unlike when they may be able to play a role as representatives in common illness. That’s fundamentally different in the context of euthanasia.’*
q2.3	pII.I	*‘The representative can be helpful in interpreting the patient’s wishes and expressions*,* but can of course never replace the request.’*
q2.4	pI.I	*‘But never that he or she [the representative] says: “I’m going to veto now.” Or*,* “on my behalf*,* it should be like this.” That’s not possible. Only a source of information for the physician.’*
q2.5	pI.II	*‘And when that clashes with what a representative believes is meant by a written advance directive… then*,* of course*,* the question is whether this is based on a reasonable argument. A physician will certainly have to heed a reasonable argument.’*
q2.6	pI.IV	*‘But then there’s also the possibility… if there’s a difference of opinion… to ask a colleague*,* like “how do they see that*,*” […] to have a second physician assess it and take that into consideration or involve the nursing team.’*
q2.7	pI.I	*‘Because*,* in addition to competence*,* undue influence also plays a role. So*,* like*,* the will can be confused […] externally by… erm*,* yes*,* undue influence […] coercion from the outside. And that’s very difficult to substantiate. But […] it happens.’*
q2.8	pII.IV	*‘I’ve seen too much of it in clinical practice*,* from representatives who don’t wish to do the right thing for those they represent. So*,* I’m very wary if the representative has conflicting interests.’*
q2.9	pI.I	*‘And I would stress that it’s useful if you [the patient] also stipulate: “if I can no longer say it myself*,* you have to ask my brother.” Because you can write down what you think yourself*,* but include in it who you [the physician] should consult.’*
q2.10	pI.I	*‘The person who wrote it must also ensure that this representative is able to do so [serving as an informant*,* providing clarification] as best as possible. Where is the document and what do I mean?’*
q2.11	pI.II	*‘I can’t imagine a physician who would say*,* “oh well*,* what those nurses are all saying about that suffering*,* hmmm*,* […] [he/she] doesn’t know the first thing about suffering.”’*
***The value of current expressions***		
q3.1	pII.III	*‘The Human Rights Convention says “own will and preferences” is important. And we need to know how to determine that.’*
q3.2	pII.III	*‘What he once stated is only interesting to us insofar as we can no longer figure out what he’s thinking.’*
q3.3	pII.II	*‘But from a legal perspective it’s very difficult*,* for… is the will still there? And that’s actually the question; we think the will get lost as dementia proceeds.’*
q3.4	pI.IV	*‘But you do ask a lot from physicians when*,* in addition to that Supreme Court ruling*,* the Euthanasia law*,* the text*,* they ask: yes*,* but European law also has frameworks that are relevant. To take those in consideration as well. You can hardly expect that from a physician.’*
q3.5	pI.II	*‘It’s good to realise behind it all that a physician who acts within Dutch law is not violating human rights.’*
q3.6	pII.III	*‘Then we come to objectively determining what is unbearable suffering. And I think you are only bound to what they say in their advance directive to a limited extent. […]. And then we just have to really consider: is it really suffering or is [the] person perhaps showing all the signs of simply being happy?’*
q3.7	pII.III	*‘We have to imagine another complicating factor: how would this patient […] experience this situation if he were competent? Because it’s not self-evident that when I describe a situation*,* […] that also means that I […] [now] feel the same as when I wrote that advance directive.’*
q3.8	pII.V	*‘Those [written advance directives] are made at a time when there’s not yet hopeless suffering at all. And then you have to discuss all kinds of hypothetical situations with your client. […] You won’t be discussing this subject in great detail for a very long time.’*
q3.9	pII.II	*‘You have a higher threshold if you make an irreversible decision that ends life. And you have a lower threshold when you have reversible and non-invasive situations.’*
q3.10	pII.II	*‘And the rotten thing is that we can’t verify it [the wish for euthanasia] anymore. And that this is becoming increasingly difficult*,* in the case of dementia*,* I mean.’*
q3.11	pII.III	*‘So*,* I think [the] wish for life […] is so primary… almost like [an] animal instinct. That if someone says “I don’t want to die*,* or not now” … that then*,* from an ethical*,* legal perspective*,* we may think that [person] is competent in the matter*,* to express their “own will and preferences” now.’*
q3.12	pII.II	*‘That’s the point I want to make: that we are interpreting much more than establishing the will.’*
q3.13	pII.II	*‘It’s always an interpretation by the listener*,* the value he attaches to the expressions.’*
***The responsibility of interpreting euthanasia requests***		
q4.1	pII.III	*‘As soon as it comes to [the assessment of the due care criteria in the context of a death wish]*,* as legal professionals we are absolutely no longer competent. It’s up to the physician to say whether the will [death wish] is there at all*,* whether the expressions [of the patient] correspond to the will [death wish]. […] The physicians must tell us: that person is not able to express [his] will [death wish]. [That] person expresses his will [death wish] extremely inconsistently. Or [his death wish is] open to multiple interpretations.’*
q4.2	pII.I	*‘But that the decision*,* the conviction that the due care criteria have been met… yes*,* that’s obviously up to the physician.’*
q4.3	pI.II	*‘In itself*,* the physician is the master of interpretation. They have the right to interpret*,* and it’s about his or her conviction.’*
q4.4	pI.III	*‘It’s also kind of straightforward. The moment you*,* as a physician*,* make your decision and are convinced*,* you are in the framework of the Euthanasia law and no other framework. You will be reviewed against that framework.’*
q4.5	pI.III	*‘That physician must be able to explain thoroughly and independently why he or she has come to his or her decision.’ […] It’s all possible*,* as long as you can explain it.’*
q4.6	pI.III	*‘The moment you have any doubts about the voluntariness or deliberateness of the request in the case of a very clear advance directive that doesn’t allow room for any misinterpretation*,* you are treading on thin ice.’*
q4.7	pI.II	*‘It’s not about a physician’s highly personal views*,* but about whether a reasonable thinking and acting physician arrives at a certain judgement in that context according to their professional standard. […] So*,* I see a point of reference in professional standards.’*
q4.8	pI.III	*‘And the same applies to such a guideline: you shouldn’t want to make it watertight*,* because it’s precisely the discretion that a physician has and the conviction that the physician should have that make it a very personal consideration for the physician.’*
q4.9	pI.II	*‘But the professional physician should have proper legal protection to act and choose in such situations.’*
q4.10	pI.IV	*‘But he [the physician] really has to support it. And if not*,* you don’t have to do it. So [I] think that protection is in place*,* but only like: is it even understood that way*,* by the physician? I wonder.’*

## Discussion

The primary objective of this study was to investigate two closely related issues: (1) how legal experts perceive that physicians can fulfill the statutory due care criteria *‘mutatis mutandis’* when they can no longer rely on ‘meaningful communication’ as a prerequisite for performing EAS, and (2) what additional legal guidance, with a particular focus on human rights aspects, can be provided to them. The analysis of the focus group discussions yielded four themes focusing on the fulfillment of the due care criteria. The first theme explores the implications of an AED. Participants acknowledged the role of AEDs under the Euthanasia law, but emphasized that patients’ current wishes should take priority. It is vital to inform patients about the limitations of their AEDs. Despite the legal standing of AEDs, the focus should remain on the patient’s current condition and expressed wishes. The second theme discusses the role of other stakeholders. According to the participants, patient representatives and healthcare professionals can assist in interpreting patients’ wishes, but cannot act as substitute decision-makers. The final decision in the EAS process rests with the physician. Conflicts between representatives and physicians should be addressed, and patients are advised to assign well-informed representatives in their AED. The third theme explores the value of current expressions. Participants discussed the challenges of recording explicit wishes in advance through AEDs due to potential changes in patients’ wishes over time. It was argued that current wishes for life should always take precedence over an AED, consistent with Human Rights Treaties [35, 36]. However, there was uncertainty about whether the current expressions of a patient with advanced dementia still accurately reflect their will. Unbearable suffering should be assessed in the current situation rather than solely based on the AED, which makes interpreting the patient’s will challenging, especially with progressing dementia. The fourth theme delves into the responsibility of interpreting euthanasia requests. There is a recognized need for interpreting both an AED and current (non)verbal expressions of the patient. Legal experts may lack the specific knowledge and competence for this task, thereby placing the responsibility on the physician. While consultation with various sources is acknowledged, participants view the physician, when appropriately supported by professional standards, as the primary authority in the decision-making process.

In line with the UNCRPD guidelines [21], patient representatives and healthcare professionals should support the patient’s decision-making process rather than replace it. However, it remains unclear whether this support extends to highly personal decisions, such as euthanasia, and to what extent. Dutch law, particularly the Euthanasia law, does not explicitly assume supportive decision-making. The Dutch Medical Treatment Contracts Act (WGBO) states that the representative must involve the patient lacking decisional capacity as much as possible [37]. Additionally, the guideline on *Decisiveness and Decision-Making Capacity* of the Long-term Care Quality Impulse Foundation (SKILZ) [38] indicates that a wish for life, even if poorly articulated, should take precedence over the AED.

Focussing on additional legal guidance, a comparison of our focus group findings with the viewpoints of legal experts who were excluded from our study, due to their prior contributions to the literature, reveals that our findings resonate with some of the views expressed by these non-participating experts. Participants agreed with Van Beers [19] and Rozemond [22, 23, 39] that current desires of patients with advanced dementia should never be disregarded and should always take precedence over previously expressed euthanasia wishes documented in an AED. This perspective challenges the traditional emphasis on precedent autonomy, which –according to Dworkin who coined this concept– prioritizes a person’s prior decisions –supposedly based on so-called critical interests grounded in rational abilities– over their current interests, which are viewed as merely experiential in nature and based on needs and emotions [40, 41]. Although the principle of precedent autonomy supports the validity of AEDs, our findings suggest that respecting a patients’ current expressions may under circumstances call for reevaluation of the AED. Additionally, Van Beers and Rozemond state that the discriminatory application of a lack of decisional capacity to prevent individuals with dementia from making their own decisions contravenes, as also indicated by our participants, not only the Human Rights Treaties [36] but also the UNCRPD [21].

Conversely, the participants also acknowledged the ethical challenges associated with interpreting the current wishes of the patient and emphasized that it is uncertain to what extent these wishes accurately reflect the patient’s genuine desire. This line of thinking shares similarities with the perspective put forth by De Bontridder [24], who argues that the evolving identity of an individual with advanced dementia should be accorded the same level of respect as their previous self, even though it is not always feasible to ascertain the specific nuances of this evolving identity. Building on these views, it is argued that, in certain instances, it may be justifiable to prioritize the patient’s wishes as documented in their AED, as their current wishes may not be fully comprehensible [24]. Although these findings reflect the ongoing ethical tension between honoring precedent autonomy and addressing current interests, they also highlight the increasing advocacy for moving away from strict adherence to precedent autonomy towards a more balanced approach that weighs both prior and current interests without a priori prioritizing critical over experiential interests [38, 41, 42]. As such, the results of our study align with those of existing research, yet they do not offer the additional legal guidance that physicians have indicated they require. Furthermore, the findings do not put forth alternative legal norms or critique the applicability of current legal standards. Physicians, while able to draw on various sources of information such as family members, patient representatives and healthcare providers, ultimately bear full responsibility for their decisions. This responsibility entails, as mentioned in the UNCRPD [43], considering both current expressions and the wishes outlined in the AED. The integration of the UNCRPD into Dutch euthanasia legislation raises questions about its application within the Dutch legal framework. There remains significant legal ambiguity regarding how the UNCRPD impacts euthanasia in the context of dementia. Some participants argue that adherence to the Euthanasia law aligns with Human Rights Treaties [35, 36], suggesting no rights violations occur as long as actions remain within legal boundaries. However, a stricter interpretation of the UNCRPD, such as that proposed in the SKILZ guideline [38], emphasizes supported decision-making and could impose limitations on the use of AEDs for euthanasia in dementia cases, potentially restricting the practice under international human rights standards. In all, the introduction of the UNCRPD necessitates a reassessment of the role of AEDs and substitute decision-making in healthcare and euthanasia decisions [22, 44], highlighting the evolving legal landscape in this field.

### Strengths and limitations

This study is the first to explore legal experts’ views on EAS in advanced dementia through focus group discussions, with a particular emphasis on physicians’ dilemmas. It represents an empirical legal study that uses focus groups to bring out common grounds among participants, offering valuable insights into the legal framework beyond the Euthanasia law and encouraging interaction among legal experts. However, we acknowledge several limitations. The study faced constraints in data saturation due to the limited number of legal experts involved and their expertise primarily in human rights, which may have resulted in missing additional relevant aspects from a broader legal perspective on the topic. Additionally, the online format of the focus groups may have influenced the dynamics of interaction, potentially limiting the depth of open discussion and the natural flow of conversations. Conversely, this format might have enhanced participants’ comfort in sharing opinions on this sensitive topic from the privacy of their own settings.

## Conclusions

From a legal perspective, the term *‘mutatis mutandis’* in Article 2.2 of the Dutch Euthanasia law sets an open norm –which is a norm that is open in substance and require further elaboration, mostly determined on a case-by-case basis to the field standards of the profession– when it comes to fulfilling the due care criteria. The physician bears the full responsibility for interpreting the AED and expressions of patients with advanced dementia. Although they do not need to go through this process in isolation, as they can rely on various sources of information, there is no additional legal guidance that can support them in arriving at a considered decision in case of euthanasia requests based on an AED. Our findings underscore that careful decision-making involves both the continuous assessment of patient’s decisional capacity and a nuanced understanding of their current wishes. The legal experts we consulted emphasized that supportive decision-making should consider both prior AEDs and current patient expressions. However, opinions differ on whether current expressions should take precedence over AEDs and on the interpretation of (non)verbal expressions.

Given these complexities, there is a clear need for further research, such as a Delphi-study, to develop comprehensive guidance for physicians. A Delphi-study could facilitate consensus among experts and provide much-needed clarity on the legal and ethical challenges surrounding euthanasia decisions for patients with advanced dementia.

## Electronic supplementary material

Below is the link to the electronic supplementary material.

Supplementary Material 1

## Data Availability

The datasets used and/or analysed during the current study are available from the corresponding author on reasonable request. Contact details for the corresponding author: D.O. Coers, Amsterdam UMC, location Vrije Universiteit Amsterdam, Medicine for Older People, De Boelelaan 1117, 1081 HV Amsterdam, The Netherlands; Amsterdam Public Health Research Institute, Aging & Later Life, Amsterdam, The Netherlands; d.o.coers@amsterdamumc.nl.

## Abbreviations

EAS: Euthanasia or physician Assisted Suicide
AED: Advance Euthanasia Directive
RDMA: Royal Dutch Medical Association
RERC: Regional Euthanasia Review Committees
UNCRPD: United Nations Convention on the Rights of Persons with Disabilities
COREQ: Consolidated criteria for reporting qualitative studies
