# Supplementary material for: A qualitative focus group study on legal experts’ views regarding euthanasia requests based on an advance euthanasia directive
Source: BMC Med Ethics. 2024 Oct 24;25:119. doi: 10.1186/s12910-024-01111-2 (PMC11515591; doi:10.1186/s12910-024-01111-2)
Supplement: Supplementary file 1 — Supplementary Material 1 [file 12910_2024_1111_MOESM1_ESM.docx]

**Appendices**

**Appendix 1 |** A description of the Dutch euthanasia procedure

| **The Dutch euthanasia procedure**  After the performance of euthanasia, the municipal pathologist is required to be notified. Then, a postmortem examination is completed and the cause of death is reported. The performing physician also reports the euthanasia to the Regional Euthanasia Review Committee (consisting of a lawyer, a physician and an ethical expert), which reviews the case and judges whether the performance was with due care. If the Regional Euthanasia Review Committee report the criteria for due care are met, no further follow-up is needed. If they conclude the criteria are not met, the case is forwarded to the Health Care Inspectorate (*‘did the physician act according to professional standards?’*) and the Public Prosecution (*‘is there a criminal liability?’*) for further investigation. Both investigations are independent, as their focus is different. If required, the Health Care Inspectorate refers the case to the Regional Medical Disciplinary Court. Following on which the case can be referred to the Central Medical Disciplinary Court. |
| --- |

**Appendix 2 |** Case description of the first euthanasia case brought to criminal court

| In 2008, the patient was diagnosed with Alzheimer’s disease. She drew up an AED in 2012 and discussed this with her general practitioner and gerontologist, which both independently determined she was competent at that time with regard to writing the AED. The AED stated she “*absolutely does not want to be placed in a nursing home for patients with dementia*” and would “*like to undergo voluntary euthanasia*” when she is “*still mentally competent and no longer able to live at home*”.  In 2015, she edited her AED by adding: *“I want to make use of the legal right to undergo euthanasia whenever I think the time is right for this… Trusting that at the time when the quality of my life has become so poor, I would like for my request for euthanasia to be honored.”.* According to her GP, she was still competent at that time^(1)^.  Cognitive decline progressed, leading to admission to a nursing home in March 2016. Her husband asked the Elderly Care Physician (ECP) to perform euthanasia based on her AED. The ECP investigated this request based on observations of the patient, speaking to her (although conversations were limited by the advanced stage of dementia) and speaking to her relatives, her former GP, her psychologist, an expert from the Euthanasia Expertise Center and other involved care professionals.  On a daily basis, the patient showed calm behavior, but also signs of severe restlessness and unhappiness. She told the nursing staff regularly that she wanted to die, but also told her ECP several times she did not yet want to die, because “*it had not gotten that bad yet*”. The ECP found that the patient was no longer capable of making decisions regarding euthanasia and eventually decided that based on her investigation and the patients’ AED, euthanasia was possible. Two independent physicians examined the case and concluded that all due care criteria were met.  In April 2016, the ECP performed the euthanasia. Beforehand, a sedative was put in the patients’ coffee to prevent agitation, as agreed upon with the family. Nevertheless, the patient tried to sit up while the lethal medication was administered and she needed to be held down by family in order to administer the remaining dose.  *Key points of the legal consequences of the case*  The ECP reported the case to the Regional Review Committee, as required by law. This committee concluded that the ECP had not met the compulsory due care criteria of a voluntary and well-considered request and exercising of due medical care. Consequently, the case was reported to the Health and Youth Care Inspectorate and the Public Prosecution Service, which respectively led to a disciplinary and criminal case.  Firstly, the Regional and Central Disciplinary Court concluded that the ECP should not have complied with the euthanasia request based on the AED, since its content was not unambiguous and the expressions of the patient with regard to the euthanasia request were inconsistent. Moreover, the ECP should have tried to discuss her intention to end the patients’ life right before administering the lethal medication. This led to a reprimand from the Regional Disciplinary Court, which was later on reduced to a warning by the Central Disciplinary Court as they found the ECP had acted reprehensibly only to a limited extend^(2, 3)^.  Secondly, the District Court acquitted the ECP of all criminal charges, stating that verbal verification of the current life- or death wish of the patient was not necessary since due to the advanced stage of dementia, given the “specific position of the incompetent patient”. Demanding this verification would undermine the aim of the AED, which is “*meant for the situation that someone writes up an AED and gets in a situation of unbearable suffering with no prospect of improvement, being no longer capable of expressing his desires*”^(4)^.  In 2020, the Supreme Court upheld the judgement of the District Court and quashed the decision of the Disciplinary Court, stating that the euthanasia request should not only be interpreted based on the actual writing of the AED, but also on the other circumstances (e.g., expressions of the patient and the beliefs of relatives and other involved care professionals) of which the physician can deduct the meaning of the patient. Furthermore, administering sedative medication beforehand does not rule out due medical care, since euthanasia should be performed in the most comfortable way for the patient^(5)^. |
| --- |

**Appendix 3** | Overview of Dutch Euthanasia law ^(6-8)^, key legislative changes and recent judicial rulings^(9-13)^

| **The Dutch Penal Code (drafted in 1881, revised in 2014)**^(14)^  According to the Dutch Penal Code, euthanasia is classified as a criminal offense, but it is treated separately from murder in Article 293. This article states that “anyone who takes another person’s life at their explicit and earnest request will be punished by imprisonment for up to 12 years,” unless the act is performed by a physician in accordance with the due care criteria outlined in the Euthanasia Act (Article 2.2) and reported to the Regional Euthanasia Review Committees (RERC).  **Legislative history**  In **1952**, a physician was prosecuted under Article 293 for ending the life of his brother, who was suffering from tuberculosis. Despite an explicit request from the patient, the physician’s appeal of force majeure was rejected, resulting in a one-year prison sentence. This case introduced the legal concept of a “conflict of duties.”  In **1973**, the Postma case initiated public debate on euthanasia in the Netherlands. A general practitioner (GP) was prosecuted for administering a lethal dose of morphine to his mother, who had requested euthanasia following a cerebral hemorrhage. The physician was found guilty for not attempting palliative care first. This case established legal conditions justifying life-shortening actions, including terminal illness, unbearable suffering, a patient's explicit request, and physician involvement.  The **1981** Wertheim case further refined these criteria, adding that euthanasia or assisted suicide decisions must involve a physician who prescribes the medication.  By **1984**, the Supreme Court ruled in the Schoonheim case that euthanasia could be permissible under certain conditions, marking the first instance where euthanasia was proven but not penalized. This case emphasized the right to self-determination, third-party assistance for a humane death, and the importance of a well-considered, explicit request.  In **the following years**, legislative proposals were introduced to remove euthanasia from the Dutch Penal Code. While the **1993** proposal to make euthanasia accountable to the Public Health Inspectorate failed, an amendment to the Funeral Care Act was passed, establishing a legal procedure for reporting euthanasia and assisted suicide cases.  In **1995**, the Chabot case extended the legal understanding of euthanasia to include patients with unbearable mental suffering. Although the psychiatrist was not penalized, the case led to the introduction of the second-opinion requirement in the due care criteria.  After further court cases, a parliamentary majority proposed new legislation in **1998**, which ultimately led to the enactment of the *Termination of Life on Request and Assisted Suicide Act*^(6)^ in **2002**. This law made the Netherlands the first country to legalize euthanasia and assisted suicide under strict conditions, including six due care criteria *(see table 1)*.  In **2002**, the Brongersma case clarified that the concept of a “completed life” did not justify euthanasia under the law. **In later years**, convictions for unreported or unreviewed cases (Van Oijen, Heringa) reaffirmed the importance of compliance with legal and ethical standards.  In **2006**, the United Nations adopted the United Nations Convention on the Rights of Persons with Disabilities (UNCRPD)^(15)^, a treaty aimed at protecting the rights and dignity of persons with disabilities. The Convention emphasizes autonomy, non-discrimination, and equal recognition before the law, which are highly relevant to the ethical debates on euthanasia for patients with advanced dementia. It calls for safeguards to protect individuals with compromised decision-making capacity, highlighting the need for careful consideration in end-of-life decisions for vulnerable groups.  In **2016**, the Netherlands ratified the UNCRPD, affirming its commitment to the rights of persons with disabilities, including those with dementia. However, the Netherlands made a reservation on Article 12, which relates to equal legal capacity. This reflects caution around substituted decision-making in cases where individuals are unable to make informed choices, such as end-of-life scenarios. The Netherlands is still seeking a balance between complying with international standards and preserving its own legal framework, including its Euthanasia law, with ongoing discussions regarding the interpretation of the Convention.  The case of Arends (**2016-2019**) brought attention to the complexities of performing euthanasia based on an advance euthanasia directive (AED) in patients with advanced dementia. The RERC^(11)^ found that essential criteria, such as a well-considered request, were not met, leading to further scrutiny. However, the Supreme Court's **2020** ruling^(9, 10)^ acquitted the physician, concluding that, in certain cases, an AED can substitute for verbal consent in patients with advanced dementia, as long as no signs of resistance are observed.  In **2021**, the Royal Dutch Medical Association (RDMA) reaffirmed in their *position paper End-Of-Life Decisions*^(16)^ that while euthanasia and assisted suicide are legally regulated, they require careful ethical consideration. The RDMA emphasized the complexity of assessing suffering in advanced dementia and advised caution when interpreting AEDs, urging physicians to consider both the patient's earlier wishes and present condition.  The **2022** revision of the RERC’s *Euthanasia Code*^(17)^ aligned with the RDMA’s position, confirming that while AEDs are valid, the evaluation of unbearable suffering and the patient's current preferences are crucial.  In **2024**, the Long-term Care Quality Impulse Foundation (SKILZ) published the *Guideline on Decisiveness and Decision-Making Capacity*^(18)^, which addresses the complexities of euthanasia in patients with dementia based on AEDs. The handbook offers an interpretation of the UNCRPD in this context, emphasizing that an AED is not an automatic substitute for a patient’s current wishes but rather serves to clarify their earlier intentions. However, this interpretation remains a topic of debate, particularly given the Dutch reservation on Article 12 of the UNCRPD, which reflects caution around decision-making capacity in cases like these. |
| --- |

**Appendix 4 |** Topic list

| Available on request. |
| --- |

**Appendix 5 |** Triangular figure of the topic list


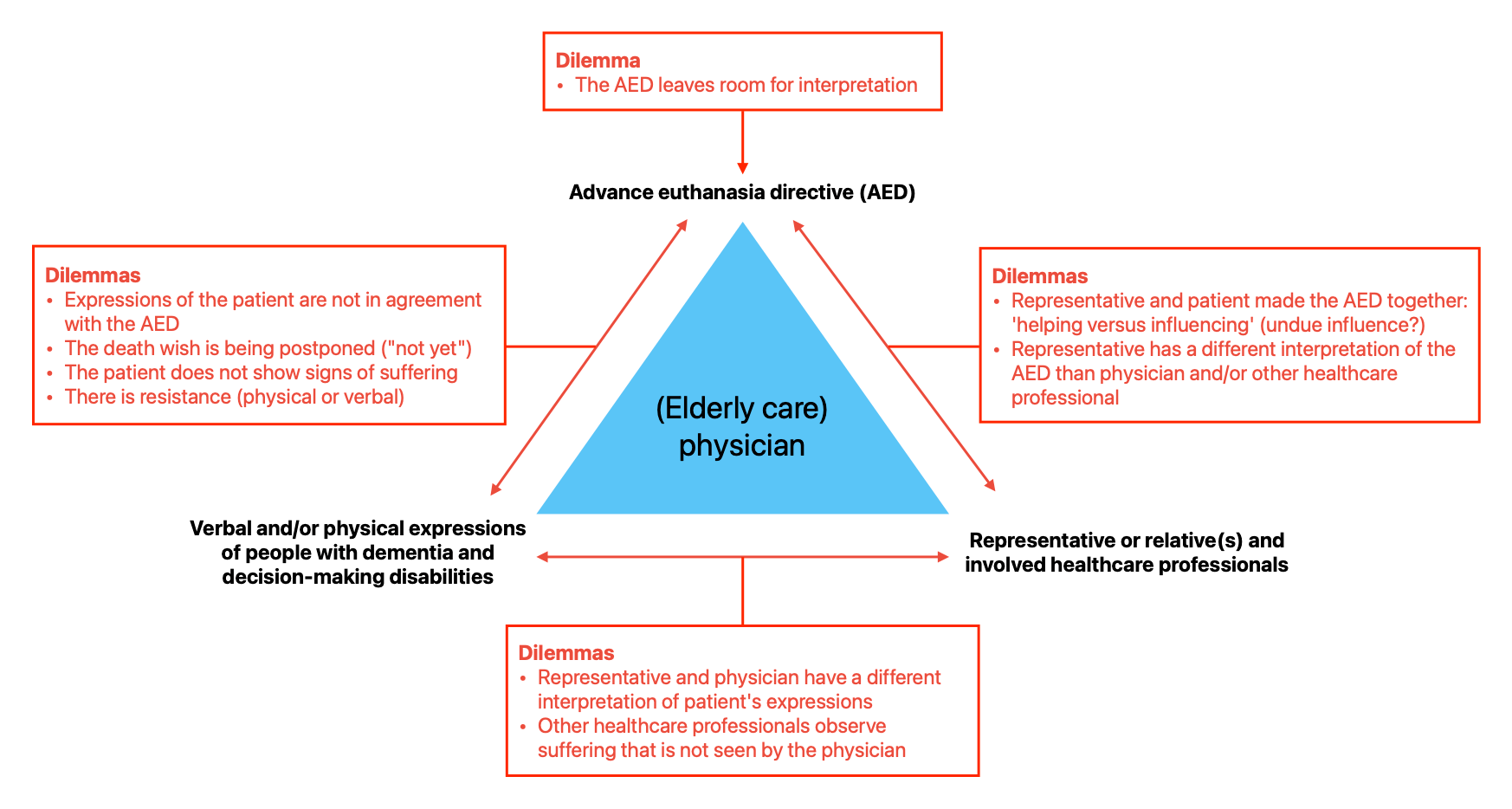


**References**

*1. Regional Euthanasia Review Committees (RERC), 2016-85.*

*2. Hearing of the Regional Disciplinary Tribunal (RTG), 24 juli 2018, ECLI:NL:TGZCTG:2019:68.*

*3. Hearing of the Central Disciplinary Tribunal (CTG), 19 maart 2019, ECLI:NL:TGZCTG:2019:68.*

*4. Hearing of The Hague District Court, 11 september 2019, ECLI:NL:RBDHA:2019:9506.*

*5. Hearing of Supreme Court, 17 december 2019, ECLI:NL:PHR:2019:1339.*

*6. Termination of Life on Request and Assisted Suicide Review Act; Article 2, paragraph 2 [in Dutch].* [*https://wetten.overheid.nl/jci1.3:c:BWBR0012410&z=2020-03-19&g=2020-03-19*](https://wetten.overheid.nl/jci1.3:c:BWBR0012410&z=2020-03-19&g=2020-03-19) *[accessed 14 June 2023].*

*7. Gevers S. Euthanasia law and practice in The Netherlands. British Medical Bulletin. 1996;52(2):326-33.*

*8. Dutch Association for Voluntary Euthanasia (NVVE). 20 jaar euthanasiewet [In Dutch]. Relevant. 2022;1:1-44.*

*9. Hearing before Supreme Court (2019, December 17, 2019).*

*10. Hearing before Supreme Court (2019, December 17, 2019).*

*11. RTE oordeel 2016-85: Hearing before the Regional Euthanasia Review Committees (RERC)(2016).*

*12. Asscher ECA, van de Vathorst S. First prosecution of a Dutch doctor since the Euthanasia Act of 2002: what does the verdict mean? J Med Ethics. 2020;46(2):71-5.*

*13. Rijksoverheid. Euthanasie en de wet: sterven met hulp van een arts [In Dutch] [Available from:* [*https://www.rijksoverheid.nl/onderwerpen/levenseinde-en-euthanasie/euthanasie*](https://www.rijksoverheid.nl/onderwerpen/levenseinde-en-euthanasie/euthanasie)*.*

*14. Dutch Penal Code [in Dutch]* [*http://wetten.overheid.nl/BWBR0001854/geldigheidsdatum_27-08-2014/afdrukken*](http://wetten.overheid.nl/BWBR0001854/geldigheidsdatum_27-08-2014/afdrukken) *[accessed 14 June 2023].*

*15. Convention on the Rights of Persons with Disabilities, 2006.*

*16. Royal Dutch Medical Association (RDMA). RDMA position: 'End of life decisions' [In Dutch]. 2021.*

*17. Regional Euthanasia Review Committees (RERC). EuthanasiaCode 2018 naar aanleiding van de arresten van de Hoge Raad d.d. 21 april 2020 inzake euthanasie bij patiënten met voortgeschreden dementie [in Dutch]. 2020.*

*18. Cees Hertogh Suzanne de Kort, Robert Helle. Handreiking Beslisvaardigheid en wilsbekwaamheid. SKILZ (Stichting Kwaliteitsimpuls Landurige Zorg); 2024. p. 108.*
